# Natural Rabies Infection in a Domestic Fowl (*Gallus domesticus*): A Report from India

**DOI:** 10.1371/journal.pntd.0003942

**Published:** 2015-07-22

**Authors:** Julie Baby, Reeta Subramaniam Mani, Swapna Susan Abraham, Asha T. Thankappan, Prasad Madhavan Pillai, Ashwini Manoor Anand, Shampur Narayan Madhusudana, Jayachandran Ramachandran, Sachin Sreekumar

**Affiliations:** 1 Chief Disease Investigation Office, Department of Animal Husbandry, Kerala, India; 2 Department of Neurovirology, WHO Collaborating Centre for Reference and Research on Rabies, National Institute of Mental Health and Neurosciences, Bangalore, India; 3 Veterinary Hospital, Koithoorkonam, Thiruvananthapuram, Kerala, India; The Global Alliance for Rabies Control, UNITED STATES

## Abstract

**Background:**

Rabies is a fatal encephalitis caused by viruses belonging to the genus Lyssavirus of the family Rhabdoviridae. It is a viral disease primarily affecting mammals, though all warm blooded animals are susceptible. Experimental rabies virus infection in birds has been reported, but naturally occurring infection of birds has been documented very rarely.

**Principal Findings:**

The carcass of a domestic fowl (*Gallus domesticus*), which had been bitten by a stray dog one month back, was brought to the rabies diagnostic laboratory. A necropsy was performed and the brain tissue obtained was subjected to laboratory tests for rabies. The brain tissue was positive for rabies viral antigens by fluorescent antibody test (FAT) confirming a diagnosis of rabies. Phylogenetic analysis based on nucleoprotein gene sequencing revealed that the rabies virus strain from the domestic fowl belonged to a distinct and relatively rare Indian subcontinent lineage.

**Significance:**

This case of naturally acquired rabies infection in a bird species, *Gallus domesticus*, being reported for the first time in India, was identified from an area which has a significant stray dog population and is highly endemic for canine rabies. It indicates that spill over of infection even to an unusual host is possible in highly endemic areas. Lack of any clinical signs, and fewer opportunities for diagnostic laboratory testing of suspected rabies in birds, may be the reason for disease in these species being undiagnosed and probably under-reported. Butchering and handling of rabies virus- infected poultry may pose a potential exposure risk.

## Author Summary

Rabies is a fatal viral disease affecting humans and other animals. Though all warm blooded animals are susceptible to this disease, rabies is commonly observed in mammals. Birds can be experimentally infected with this virus; however, naturally occurring rabies infection in birds has been reported very rarely. We report an unusual case of natural rabies infection in a domestic fowl from India. The bird was bitten by a stray dog and succumbed after a month. The brain tissue from the carcass was tested at a laboratory and was found to be positive for rabies virus antigens. This report indicates that rabies is a disease that can affect birds. Most often birds succumb due to shock or complication of animal bite injury and may not survive until the development of clinical signs of rabies infection. Moreover, fewer opportunities for diagnostic laboratory testing of suspected rabies in a bird may be a reason for the disease in these species being underestimated. Butchering and handling of virus-infected poultry may pose a potential biohazard.

## Introduction

Rabies is a fatal encephalitis caused by the viruses belonging to the genus *Lyssavirus* of the family Rhabdoviridae. It is a viral disease primarily affecting mammals, though all warm blooded animals are susceptible. In India and other Asian countries more than 90% of human infections occur due to exposure to rabid dogs, while cats, monkeys and other wild animals are reported to transmit the infection in the rest of the cases **[1].** Perusal of available literature revealed that natural infection of birds with rabies virus has been documented uncommonly. The occurrence of rabies in a chicken under natural conditions is considered extremely rare **[2].** The present case of clinical rabies in a domestic fowl was identified from an area which has a significant stray dog population and is highly endemic for canine rabies.

## Methods

### Ethics statement

This study included samples received for diagnostic confirmation from a domestic fowl that died naturally. No tissue or clinical sample was obtained from the dead fowl specifically for the purpose of this study. No human subject or human clinical samples were included in this study. Hence, ethical clearance from the institutional review boards (IRB, NIMHANS and IRB, CDIO) was not required.

The carcass of a domestic fowl (*Gallus domesticus*) was brought to the rabies diagnostic laboratory at the Chief Disease Investigation Office, Department of Animal Husbandry, Kerala, India for diagnosis of rabies. The bird had been bitten by a stray dog one month back in its breast muscle. The wound was treated locally. After one month, the bird appeared droopy and off-feed for a day and succumbed. As rabies was frequently reported in the locality among dogs and other domestic animals, the owner brought the carcass for ruling out rabies. The stray dog that bit the fowl could not be traced and laboratory confirmation of its rabid status could not be done.

### Sample collection

A detailed necropsy was conducted. The whole brain was collected from the bird with all biosafety precautions. Two independent laboratories with facilities for rabies diagnosis tested the sample.

### Laboratory tests

Touch impressions of the brain were stained by Seller’s stain and examined for the presence of Negri bodies. Impressions, fixed in high grade chilled acetone at -20°C, were subjected to the fluorescent antibody test (FAT) for detection of rabies virus nucleoprotein antigens **[3]**. A one step TaqMan real time PCR targeting the nucleoprotein gene was carried out as described earlier **[4]** on the brain tissue for confirmation of rabies virus infection. Partial gene sequencing was carried out by amplifying a 446 bp region in the nucleoprotein gene using nested PCR **[5].** PCR products were purified using a commercial kit (QIAquick Gel purification kit, Qiagen, UK) and custom sequenced from Amnion Biosciences Pvt. Ltd, Bangalore, India using gene specific primers. The sequence was deposited in the GenBank database under accession number KP316199. Partial nucleoprotein gene sequences of rabies virus isolates from GenBank representing various geographical regions in India and two other countries from the Indian subcontinent i.e Sri Lanka and Nepal were used to investigate the phylogenetic relationship with the present rabies virus strain. The sequences were aligned using ClustalW, and a maximum likelihood phylogeny tree was constructed using MEGA5 software **[6]** with bootstrap replication values of 1000.

## Results

No significant lesions could be observed in any of the visceral organs on post-mortem examination. The dog bite wound on the breast muscle was fully healed. The brain impressions tested positive for rabies virus antigens by FAT, however Negri bodies could not be demonstrated. The brain tissue sample was positive for rabies viral RNA by TaqMan real time PCR.

**Fig 1:**
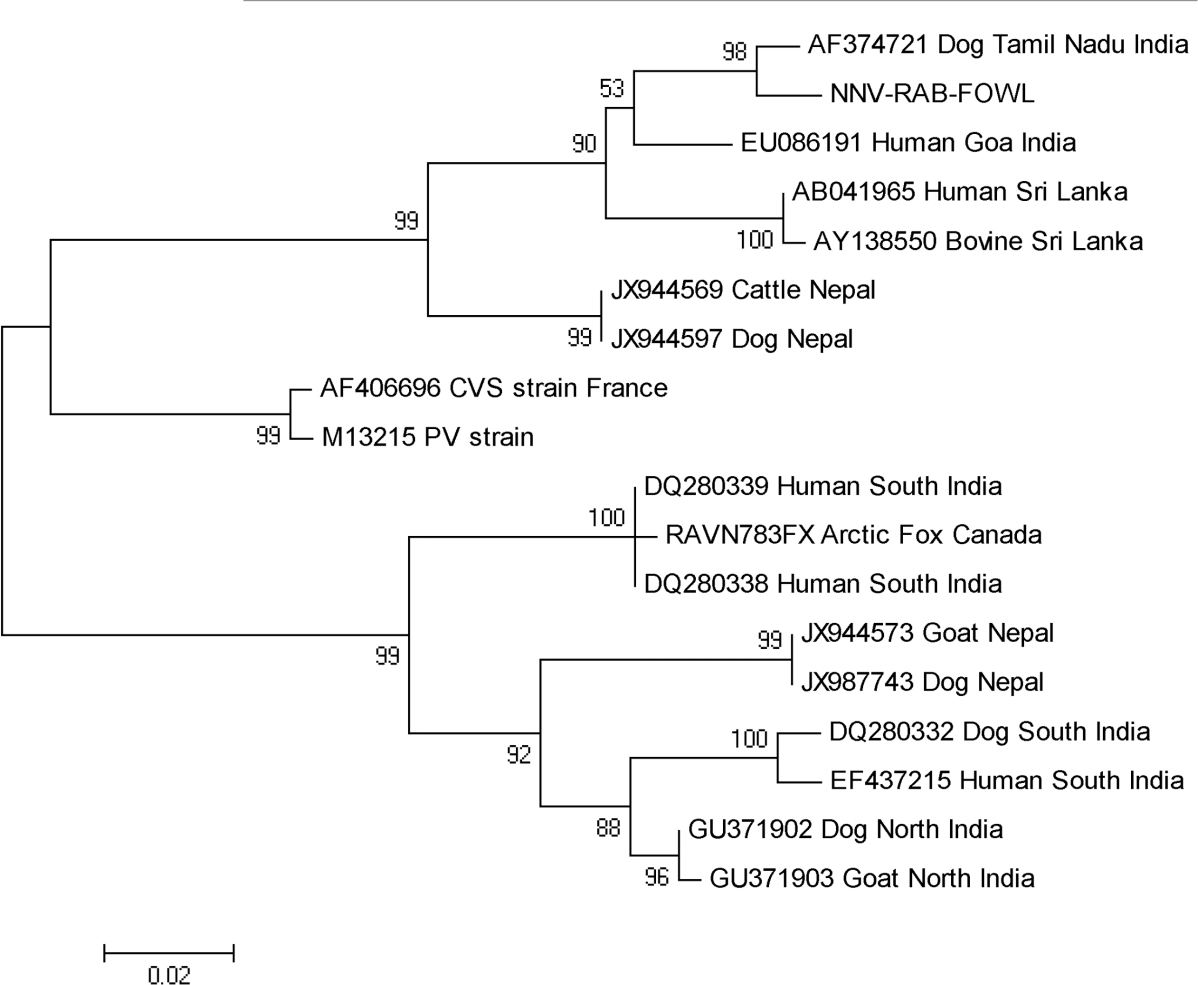
Phylogenetic tree. Maximum likelihood phylogenetic tree was constructed with MEGA 5 software using nucleoprotein gene sequences of rabies virus isolates from GenBank representing various geographical regions in India and two other countries from the Indian subcontinent. Bootstrap replication values of 1000 were used and the numbers below the branches indicate the percentage bootstrap support for each cluster. Reference strains (M13215 PV strain and AF406696 CVS strain France) and Arctic Fox Canada strain (RAVN783FX) have been included for comparison.

The phylogenetic tree comprising of various nucleoprotein gene sequences of rabies virus isolates from India can be divided into 2 divergent clusters **(Fig 1)**. The lower cluster comprises of most of the isolates from the northern and southern part of India belonging to the Arctic/Arctic-like lineage, along with the Arctic Fox isolate from Canada (included for comparison). The upper cluster includes the present strain (NNV-RAB-FOWL) and other rabies virus strains from Southern India, Sri Lanka and Nepal which belong to the distinct Indian subcontinent lineage.

## Discussion

Rabies has been conventionally considered as a disease of mammals and clinical cases of naturally acquired rabies has been reported in birds infrequently. A few anecdotal reports were published in the late 1950’s, indicating the rare occurrence of rabies in birds **[7, 8]**; however they remain uncorroborated by lack of additional reports with robust laboratory evidence of naturally acquired rabies in birds. Gough and Jorgenson (1976) examined 343 serum samples of birds for antibodies against rabies virus and found that 23 of them had low passive haemagglutination titers **[9].** However, in another serological survey in captured birds, no significant titers were detected **[10].** A variety of birds have been infected experimentally with development of clinical signs, often without development of neurological features, or with recovery from clinical signs **[2,11].**

The present report indicates that rabies is a disease that can affect birds. Lack of obvious clinical signs and fewer opportunities for diagnostic laboratory testing of suspected rabies in a bird, may be the reason for the disease in these species being undiagnosed and probably underestimated. The locality of the bird in the present case is highly endemic for rabies **[12]** and the presence of an easily accessible diagnostic facility may be the reason the disease could be identified in poultry. Most often birds succumb due to shock or complication of animal bite injury and may not survive until the development of clinical rabies infection.

Phylogenetic analysis based on partial nucleoprotein gene sequencing revealed 98% homology of the present rabies virus strain (NNV-RAB-FOWL) to a canine rabies virus isolate (AF374721) from Chennai, in Tamil Nadu state in south India. Interestingly, it clustered with another Indian isolate from Goa, a southern state and also with distinct isolates from Sri Lanka, rather than with other Indian isolates in the Arctic/Arctic-like virus cluster, the extensive circulation of which has been reported from India. Earlier reports had speculated the presence of this distinct Sri Lankan variant in India and suggested that movement of humans and their animals between Sri Lanka and India, particularly within the southeastern coastal area of the mainland, may have resulted in the movement of this variant between these geographically separate regions **[13–15].** Other studies confirmed the circulation of this distinct variant found only in Sri Lanka and mostly the southern part of India **[16].** However, recently isolates from Nepal have also been reported to be phylogenetically related to these distinct variants and this lineage has been designated as the Indian subcontinent rabies virus clade **[17].** This clade with phylogenetically related strains from South India (including the present strain), Sri Lanka and Nepal is also evident in **Fig 1.** Sequencing and phylogenetic analysis of additional isolates representative of various geographical areas in India and other countries in the Indian sub-continent may aid in further elucidation of the epidemiological significance of these variants.

In conclusion, a case of naturally acquired rabies infection in a bird species, *Gallus domesticus* is reported in India for the first time. It indicates that spill over of infection even to unnatural hosts is possible in highly endemic areas. The risk of exposure through consumption of infected meat, though unlikely, and butchering/handling of rabies virus-infected poultry can potentially pose a risk of transmission of rabies to humans, although never reported to date.
